# Highly Sensitive Detection of Chymotrypsin Based on Metal Organic Frameworks with Peptides Sensors

**DOI:** 10.3390/bios13020263

**Published:** 2023-02-13

**Authors:** Lei Liu, Cheng Liu, Li Gao

**Affiliations:** 1 Department of Kidney Transplantation, The Second Xiangya Hospital of Central South University, Changsha 410011, China; 2 School of Life Sciences, Jiangsu University, Zhenjiang 212013, China

**Keywords:** chymotrypsin, peptide, CuNCs, limit of detection

## Abstract

In this study, peptides and composite nanomaterials based on copper nanoclusters (CuNCs) were used to detect chymotrypsin. The peptide was a chymotrypsin-specific cleavage peptide. The amino end of the peptide was covalently bound to CuNCs. The sulfhydryl group at the other end of the peptide can covalently combine with the composite nanomaterials. The fluorescence was quenched by fluorescence resonance energy transfer. The specific site of the peptide was cleaved by chymotrypsin. Therefore, the CuNCs were far away from the surface of the composite nanomaterials, and the intensity of fluorescence was restored. The limit of detection (LOD) using Porous Coordination Network (PCN)@graphene oxide (GO) @ gold nanoparticle (AuNP) sensor was lower than that of using PCN@AuNPs. The LOD based on PCN@GO@AuNPs was reduced from 9.57 pg mL^−1^ to 3.91 pg mL^−1^. This method was also used in a real sample. Therefore, it is a promising method in the biomedical field.

## 1. Introduction

Chymotrypsin, one of the serine proteases, is involved in a number of physiological processes, including necrosis and apoptosis [1]. It participates in the pathogenesis of various diseases [2,3]. At present, researchers have developed certain methods to detect the activity of chymotrypsin. Colorimetric reaction detection [4], high performance liquid chromatography (HPLC) [5], mass spectrum (MS) [6,7], electrochemical detection [8], and surface plasmon resonance (SPR) detection [9], etc., were used for chymotrypsin. However, the detection sensitivity in certain methods needs to be improved. A number of methods require specific equipment. Developing a sensitive detection method of chymotrypsin is necessary [10]. A fluorescence spectrometer is simpler than certain more complicated instruments, such as HPLC and MS. Fluorescence resonance energy transfer (FRET) for chymotrypsin detection based on quantum dots [11], gold nanoparticles [12], and fluorescein–rhodamine B fluorophore pairs [13,14] is a low cost method. However, the detection sensitivity of this method still needs to be improved.

A metal organic framework (MOF) has a large surface area and contains uniform porous crystal material with unique structure and flexible porosity [15,16,17,18]. Certain metal ions and organic ligands show adjustable fluorescence properties. Changes in the structure and properties of MOFs will result in changes in fluorescence. Therefore, MOF-based fluorescence sensors have received a substantial amount of attention. A series of multi-dimensional MOFs have been prepared as fluorescent platforms for biological sensing [19,20,21,22]. Porous coordination network (PCN) is a kind of porous metal organic framework material. Hybrid materials can achieve superior efficiency by eliminating certain shortcomings [21]. In this study, PCN@GO@AuNP composite nanomaterials were used for fluorescence quenching. Copper nanoclusters (CuNCs) are not only similar to noble metal nanoclusters in fluorescence properties, particle size, and biocompatibility, but also stable and cheap [23,24,25,26,27,28]. Therefore, CuNCs offer great potential for development and application [29,30,31,32,33,34,35,36]. On the basis of the aforementioned studies, CuNCs and composite nanomaterials were used to detect chymotrypsin. A low-cost, highly sensitive detection method of chymotrypsin involving simple operation was developed.

## 2. Materials and Methods

### 2.1. Chemical Reagents and Instruments

The peptide sequence, RRHFFGCEKEKEKEPPPPC [12,37], was obtained from Sangon (Shanghai, China) Co., Ltd. The chymotrypsin was obtained from Shanghai Linc-Bio Science (Shanghai, China) Co., Ltd. Cu(NO_3_)_2_, His, Ascorbic Acid, NaBH_4_, and other chemicals were obtained from the National Pharmaceutical Group Chemical Reagent (Beijing, China) Co., Ltd. PCN was obtained from Xi’an Qiyue Biotechnology(Xi’an, China) Co., Ltd. Thrombin, Lysozyme, N-Hydroxysuccinimide (NHS), N-(3-Dimetylaminopropyl)-N′-etylcarbodiimid (EDC), IgG, and BSA were obtained from Sigma-Aldrich (Shanghai, China). The serum was bought from Tianhang Biotechnology Co., Ltd. (Zhejiang, China). VEGF, a tubular dialyzer, and a 0.22 μm filter were obtained from Shanghai Sangon Co., Ltd. The intensity of the fluorescence was obtained using a Bio-Tek Synergy H_4_ multifunctional microplate reader. 

### 2.2. Methods

**Figure 1:**
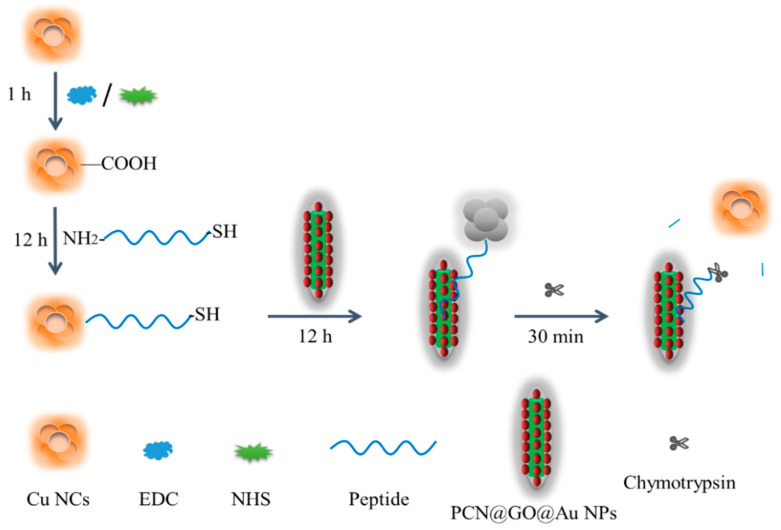
Scheme of chymotrypsin detection using copper nanoclusters and composite nanomaterials.

The PCN@AuNP@GO nanohybrid was prepared using GO layer coated onto the surface of PCN@AuNP based on strong π–π stacking and hydrogen bond interactions. Then, PCN@GO@AuNP and PCN@AuNP composite nanomaterials were used to quench fluorescence. Copper nanoclusters were prepared and activated by EDC and NHS. The amino group at one end of the peptide reacted with the carboxyl group at one end of the activated copper nanoclusters [38]. Then, the fluorescence of copper nanoclusters was quenched with hybrid materials, PCN@AuNPs@GO or PCN@AuNPs, based on FRET. The peptide with the fluorescence can covalently bind to the surface of the AuNPs of PCN@GO@AuNP and PCN@AuNP composite nanomaterials. The fluorescence was quenched by composite nanomaterials. Chymotrypsin cleaved the peptide. The target peptide (RRHFFGCEKEKEKEPPPPC) with alternating amino acid residues of negatively charged glutamic acid (E) and positively charged lysine (k) has attracted more and more attention due to its antifouling properties. Functional peptides can resist the non-specific adsorption of charged proteins on the surface of the sensor [39]. The fluorescence group will be far away from the surface of the composite nanomaterials. Therefore, the intensity of fluorescence was improved after chymotrypsin was added [14]. Figure 1 shows the principle of detection. Chymotrypsin can be detected with the change in fluorescence intensity after chymotrypsin was added.

**Figure 2:**
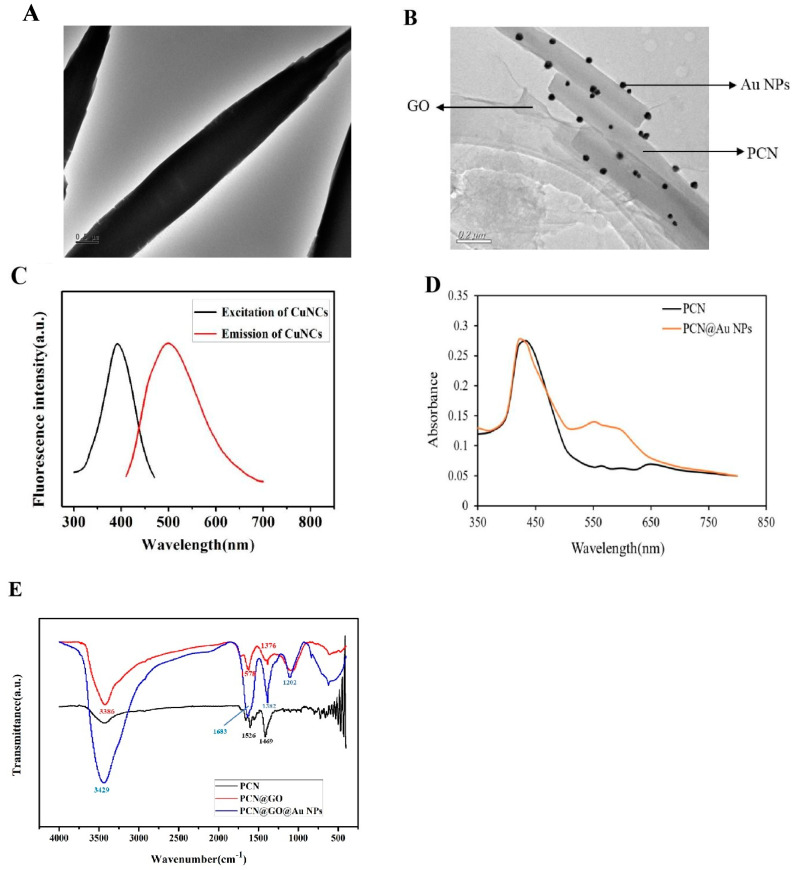
(**A**) PCN nanomaterial and TEM. (**B**) PCN@GO@AuNP composite nanomaterial and TEM. (**C**) Fluorescence excitation and emission spectra of CuNCs. (**D**) PCN@AuNP composite nanomaterial. (**E**) The peaks of hydroxy O-H were 3386 and 1376 cm^−1^, and 1578 cm^−1^ was the absorption peak of epoxy C-O. These were the characteristics of GO. After AuNPs were added, the peak moved; it showed that AuNPs bound to the surface of PCN@GO in the complex.

An amount of 100 μL PCN (0.1 mg mL^−1^) was mixed with 2 mL GO solution (0.25 mg mL^−1^). Under the assistance of NH_2_NH_2_·H_2_O, PCN@GO composite was formed under ultrasonic wave. Then, the final concentration of 0.1 mg mL^−1^ of HAuCl_4_ PCN@GO in solution was reached. The resulting mixture was then stirred, and 25 μL NaBH_4_ solution (3.8 mg mL^−1^) was added. The prepared mixture was washed twice with water and centrifuged at 4500 rpm for 10 min to remove the unreacted chemicals. The mixture was then subjected to ultrasonic treatment at 60 °C for 72 h. Then, excess chemicals were removed from PCN@GO@AuNP composite nanomaterials. Then, it was dispersed in water (Figure 2A,B). PCN@AuNPs were prepared by adding 80 μL HAuCl_4_ (10 mg mL^−1^) into the final concentration of 0.1 mg mL^−1^ PCN solution. Then, 25 μL NaBH_4_ solution (3.8 mg mL^−1^) was added to the resulting mixture; thus, the resulting mixture was stirred. The resulting mixture was then washed twice with water and centrifuged at 4500 rpm for 10 min to remove the unreacted chemicals. The obtained PCN@Au NP nanomaterials were dispersed in water for standby (Figure 2C).

CuNCs were synthesized via an improved method. In short, 360 μL of 10 mM Cu(NO_3_)_2_ solution was mixed with 8.0 mL of 100 mM histidine solution. Then, 400 μL of 100 mM ascorbic acid solution was added. The impurities were removed from the CuNCs stabilized by histidine using a dialysis tube (MWCO 1000 Da). The obtained CuNC solution was filtered using a 0.22 μM filter. Next, the carboxyl group on the surface of the CuNCs was activated with 1 mL CuNC solution mixed with 1 mg EDC and 0.5 mg NHS for 1 h. The solution of 1 mL CuNCs with the activated carboxyl group was reacted with 200 μL peptide (6 μM) overnight. The carboxyl group at one end of the CuNCs and the amino group at the other end of the peptide were covalently combined to form the peptide with a fluorescent group. The CuNC-P (peptide) solution was prepared for use. The volume of the reaction system was 400 μL. The supernatant was removed via centrifugation at 12,000 rpm for 20 min. The ultrapure water was added to the 400 μL of the reaction system. The fluorescence intensity was recorded. Then, chymotrypsin was added. The CuNCs were excited at 390 nm, and the emission wavelength ranged from 410 nm to 650 nm with increments of 2 nm (Figure 2C). As shown in Figure 2D, the absorbance spectrum was the same as that reported by Huang et al., which proved that the material was successfully prepared.

## 3. Results

### 3.1. Preparation of PCN@AuNPs

**Figure 3:**
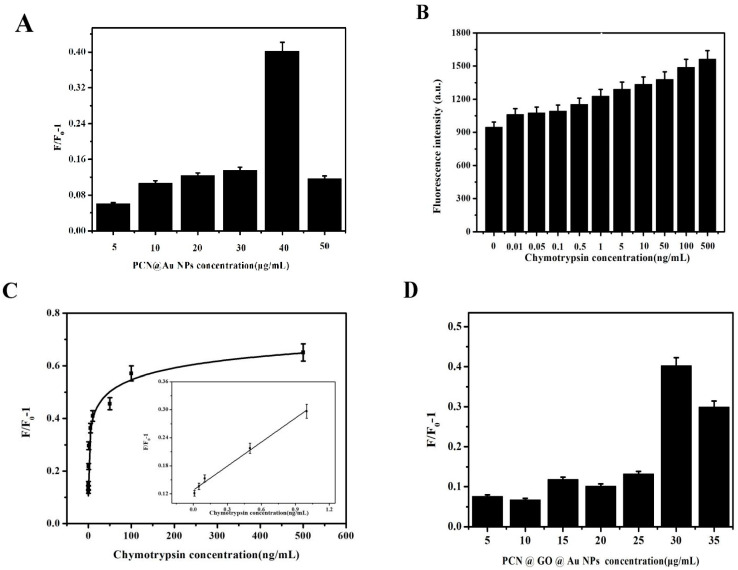
(**A**) The fluorescence intensity caused by different concentrations of PCN@AuNPs. (**B**,**C**) Detection of different concentrations of chymotrypsin using Cu NC-P sensors. (**D**) The fluorescence intensity with different concentrations of PCN@GO@AuNP composite nanomaterials. (The sample number was 5).

In total, 40 μL CuNC-P solution was added to the system; then, different concentrations of CuNC-P were added to the PCN@AuNPs composite nanomaterial solution (5, 10, 20, 30, 40, and 50 μg mL^−1^). The intensity of fluorescence was F_0_. Then, 10 ng mL^−1^ chymotrypsin was added, and the fluorescence intensity was measured as F after 30 min. The change in fluorescence intensity was F/F_0_-1. This is shown in Figure 3A. The change in fluorescence intensity was the maximum at the concentration of 40 μg mL^−1^ NPs. In total, 40 μg mL^−1^ PCN@AuNPs was selected for use in the following experiments.

### 3.2. Sensitive Detection Based on PCN@Au NPs

In total, 40 μL CuNC-P solution was added into the system with the same concentration PCN@AuNPs. The intensity of fluorescence was F_0_. Chymotrypsin (10, 50, 100, 500 pg mL^−1^; 1, 5, 10, 50, 100, 1, and 500 ng mL^−1^) was added. After 30 min, the intensity of fluorescence was F. The results are shown in Figure 3B. The change in fluorescence intensity was linear with a low concentration of chymotrypsin. The change in fluorescence intensity caused by concentrations from 0.01 to 1 ng mL^−1^ chymotrypsin was linear. The linear regression equation was y = 0.1366x + 0.0710, R^2^ = 0.96. The limit of detection (LOD) based on 3S/N was 9.57 pg mL^−1^.

### 3.3. Preparation of PCN@GO@Au NPs

Different concentrations of CuNC–P were added to PCN@GO@AuNPs. The fluorescence intensity of the composite nanomaterial solution (5, 10, 15, 20, 25, 30, and 35 μg mL^−1^) was F_0_. Then, 10 ng mL^−1^ chymotrypsin was added, and the fluorescence intensity was measured as F after 30 min. The change in fluorescence intensity was F/F_0_-1, which is shown in Figure 3D. When the concentration of NPs was 30 μg mL^−1^, the change in fluorescence intensity was the maximum. An amount of 30 μg mL^−1^ PCN@GO@AuNP composite nanomaterials was used in the following experiments.

### 3.4. Kinetic Analysis Based on PCN@GO@AuNPs

**Figure 4:**
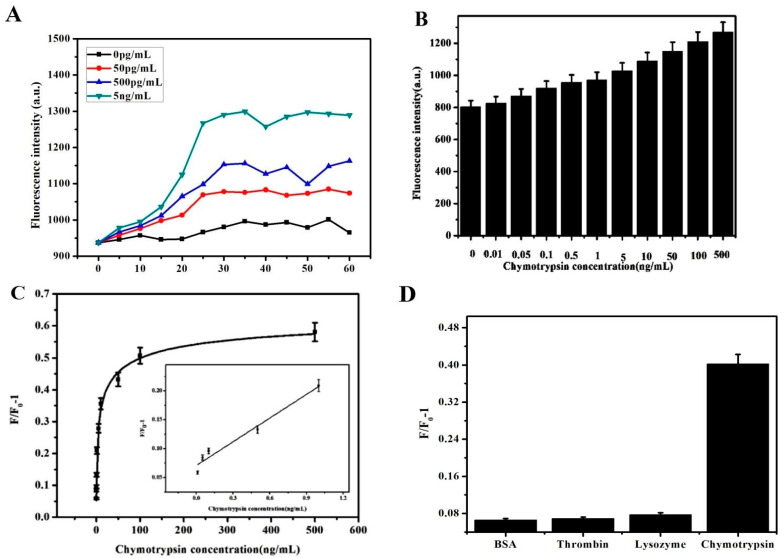
(**A**) The fluorescence intensity of the sensor with three kinds of concentrations (50, 500, and 5 ng mL^−1^) of chymotrypsin with different time. (**B**,**C**) Detection of chymotrypsin using CuNC–peptide sensors. (**D**) The intensity of fluorescence caused by 10 ng mL^−1^ IgG, chymotrypsin, thrombin, VEGF, BSA, and lysozyme. (The sample number was 5).

In total, 40 μL CuNC–P solution was added into the reaction system. Then, 30 μg mL^−1^ PCN@GO@AuNP composite nanomaterial was also added. The final concentrations of the two were the same. Three different concentrations of chymotrypsin (50, 500, and 5 ng mL^−1^) were added to measure F/F_0_-1 at different times. Figure 4A shows the results. The intensity of the fluorescence increased rapidly in 20 min. The change in fluorescence intensity for three concentrations of chymotrypsin was relatively stable at 30 min. Therefore, 30 min was selected as the reaction time.

### 3.5. Sensitive Detection Based on PCN@GO@Au NPs

Different concentrations of chymotrypsin (10, 50, 100, and 500 pg mL^−1^; 1, 5, 10, 50, 100, and 500 ng mL^−1^) were added into the reaction system for 30 min. Figure 4B,C show the results. The change in fluorescence intensity increased linearly with the low concentration of chymotrypsin. The fluorescence intensity of the sensor increases with the increasing concentration of chymotrypsin. Then, it was gradually stable. The fluorescence intensity from 0.01 to 1 ng mL^−1^ chymotrypsin was linear with the concentration. The equation of linear regression was y = 0.1712x + 0.1281, R^2^ = 0.99. The LOD was 3.91 pg mL^−1^. Compared to PCN@AuNP composite nanomaterials, the sensitivity of the sensor was improved. As shown in Table 1, the LOD in this method was lower. The lower LOD also showed that this method had higher sensitivity and also confirmed that hybrid materials can achieve superior efficiency. 

### 3.6. Selective Analysis Based on PCN@GO@Au NPs

Chymotrypsin, thrombin, VEGF, IgG, BSA, and lysozyme (every protein was 10 ng mL^−1^) were added, respectively. The reaction time was 30 min. Figure 4D shows the results. The change in fluorescence intensity caused by chymotrypsin was the maximum because of the specific and stronger binding between aptamer and chymotrypsin compared to other proteins.

### 3.7. Application in Real Sample

In order to prove the stability of the sensor and its practical application value, three concentrations of chymotrypsin (10, 50, and 100 pg mL^−1^) were used in the serum. As shown in Table 2, the range of recovery was from 96.02% to 110.04%. The relative standard deviation was from 1.41% to 2.43%. These results showed that this sensor can meet the requirements of application.

## 4. Conclusions

A detection method of chymotrypsin was developed using copper-nanocluster-labeled peptides and composite nanomaterials. First, CuNCs were made. EDC and NHS were added to activate their carboxyl group. Then, peptides were added. The carboxyl group combined with the amino group at one end of the peptide to form the peptide label with fluorescence. Then, the prepared PCN@GO@AuNP and PCN@AuNP composite nanomaterials were used for fluorescence quenching. The thiol group at the other end of the peptide can covalently combine with the composite nanomaterial. The fluorescence was quenched. Then, chymotrypsin was used to cleave the peptide, and the fluorescence intensity was restored. Chymotrypsin could be detected with the change in fluorescence intensity before and after adding chymotrypsin. The detection limit of the AuNP sensor was obviously lower than that of using PCN@AuNPs. The LOD was reduced from 9.57 pg mL^−1^ to 3.91 pg mL^−1^. This reduced LOD showed that the “three in one” composite nanomaterials had an excellent effect and performance in the detection of chymotrypsin. In this method, the physical adsorption was removed with centrifugation, and the false positive signal was reduced. Furthermore, the target peptide with alternating amino acid residues of negatively charged glutamic acid (e) and positively charged lysine (k) can also resist the non-specific adsorption of charged proteins on the surface of the sensor. The synthesis cost of CuNCs was low and stable. At the same time, the composite material was introduced to improve the performance of the sensor and the sensitivity of detection, which provided a method for the detection of chymotrypsin.

## Figures and Tables

**Table 1:** Results for the detection of chymotrypsin.

Method	Analyst	Detection Limit	Reference
Fluorescence detection	AuNPs/peptide	0.095 ng mL^−1^	[12]
Fluorescence detection	Fluorophore/peptide	10 ng mL^−1^	[40]
Fluorescence detection	Colloidal GO	0.0475 ng mL^−1^	[41]
Fluorescence detection	carbon dots	0.3 ng mL^−1^	[42]
Fluorescence detection	GO/up-conversion nanoparticles	7.9 pg mL^−1^	[43]
Fluorescence detection	Ratiometric fluorescence probe	8.4 ng mL^−1^	[44]
Fluorescence detection	GO/dC12-AgNCs	3 ng mL^−1^	[45]
Fluorescence detection	PCN@GO@AuNPs/CuNCs	3.91 pg mL^−1^	This study

**Table 2:** The detection of chymotrypsin in the serum (*n* = 3).

Samples	Added (pg mL^−1^)	Obtained (pg mL^−1^)	Recovery (%)	RSD (%)
1	0	0	--	--
2	10	11.004	110.04	1.92
3	50	48.012	96.02	2.43
4	100	103.524	103.52	1.41

## Data Availability

Not applicable.
